# Real-Time Volume-Rendering Image Denoising Based on Spatiotemporal Weighted Kernel Prediction

**DOI:** 10.3390/jimaging11040126

**Published:** 2025-04-21

**Authors:** Xinran Xu, Chunxiao Xu, Lingxiao Zhao

**Affiliations:** 1 School of Biomedical Engineering, Division of Life Sciences and Medicine, University of Science and Technology of China, Hefei 230026, China; xuxinran@mail.ustc.edu.cn (X.X.); feimos@mail.ustc.edu.cn (C.X.); 2 Suzhou Institute of Biomedical Engineering and Technology, Chinese Academy of Sciences, Suzhou 215613, China

**Keywords:** ray tracing, volume rendering image denoising, realistic volume rendering

## Abstract

Volumetric Path Tracing (VPT) based on Monte Carlo (MC) sampling often requires numerous samples for high-quality images, but real-time applications limit samples to maintain interaction rates, leading to significant noise. Traditional real-time denoising methods use radiance and geometric features as neural network inputs, but lightweight networks struggle with temporal stability and complex mapping relationships, causing blurry results. To address these issues, a spatiotemporal lightweight neural network is proposed to enhance the denoising performance of VPT-rendered images with low samples per pixel. First, the reprojection technique was employed to obtain features from historical frames. Next, a dual-input convolutional neural network architecture was designed to predict filtering kernels. Radiance and geometric features were encoded independently. The encoding of geometric features guided the pixel-wise fitting of radiance feature filters. Finally, learned weight filtering kernels were applied to images’ spatiotemporal filtering to produce denoised results. The experimental results across multiple denoising datasets demonstrate that this approach outperformed the baseline models in terms of feature extraction and detail representation capabilities while effectively suppressing noise with superior performance and enhanced temporal stability.

## 1. Introduction

In recent years, denoising techniques in real-time realistic rendering have become a pivotal topic in the rendering field. Denoising images generated by Direct Volume Rendering (DVR) can quickly reconstruct clear and accurate rendered images or videos, which is crucial for the interactive exploration of volumetric data. While Monte Carlo (MC) ray-tracing methods can produce high-quality rendering results, they require substantial time costs due to the need for extensive sample calculations for each pixel [1]. In real-time rendering scenarios, to meet the demands of interactive applications, rendering software must achieve frame rates exceeding 30 frames per second (fps). This requirement necessitates the emission of rays at an extremely low samples per pixel (spp) rate for each pixel. The low sampling rate inevitably introduces significant random noise, severely influencing the presentation of image contours, shadows, and other detailed features [2]. Consequently, the development of efficient techniques for the rapid reconstruction of high-fidelity rendered images represents an area of considerable research significance.

With the advancement of deep learning, an increasing number of denoising solutions employ lightweight neural networks to approximate noise-free image reconstruction. These methods are primarily designed for surface-rendering tasks, utilizing noise-free geometry buffers (G-buffers) generated during the rendering process as auxiliary features to guide the denoising process. Based on the output type of denoising networks, these methods can be classified into radiance prediction and kernel prediction [3]. Radiance-prediction methods directly fit the noise-free radiance value for each pixel through neural networks, such as Feature Dual Autoencoder (FDAE) [4] and NVIDIA OptiX AI-accelerated Denoiser (ONND) [5]. Kernel-prediction methods predict filter kernels through neural networks and apply them pixel-by-pixel for filtering and denoising, such as Kernel-Predicting Convolutional Networks (KPCNs) [6] and Weight Sharing Kernel Prediction (WSKP) [7]. The G-buffers in surface rendering contain noise-free rendering features (e.g., albedo, normals, world coordinates, and depth) that provide effective object edge and detail information for denoisers. However, surface-rendering techniques are typically suitable for scenes with well-defined surface contours. In medical visualization tasks, DVR technology is often employed to render objects without clearly calculable surface boundaries, such as soft tissues, blood vessels, and smoke structures. DVR images exhibit higher noise levels, and the G-buffers contain noise [8]. This limits the performance of surface-rendering denoisers when processing DVR images, making the design of effective denoising strategies more challenging. Additionally, there are relatively few denoisers specifically designed for DVR images.

DVR denoising faces three main challenges. First, the high noise levels in Volumetric Path Tracing (VPT)-based DVR images make it difficult to extract effective feature information. Since G-buffers generated by volume rendering typically contain noise and lack accurate surface intersection points, current MC denoising algorithms tend to produce blurry and darkened results when applied to DVR outputs, making it difficult to reconstruct clear material structures [9]. Second, to achieve clearer denoising results, denoisers need to effectively establish the relationships between G-buffers and rendering outputs, which typically requires a large number of trainable parameters, significantly increasing the computational time during inference. Furthermore, volume-rendering applications often demand the real-time interactive exploration of complex structures, placing higher demands on the temporal stability of rendering results. Therefore, developing a lightweight and efficient temporal denoising network is crucial.

To address these challenges, we propose a novel spatiotemporal denoising framework for realistic volume rendering that achieves real-time performance while delivering high-quality image reconstruction and robust temporal stability. Our denoiser includes two stages: Feature extraction and kernel prediction. In the first stage, we employed the reprojection algorithm to derive temporal information from historical frames, and integrated temporal filtering kernels to dynamically adjust the per-pixel weights during temporal fusion. In the second stage, we introduced a dual-stream convolutional encoder–decoder architecture. This design processes radiance features and G-buffers from both historical and current frames as separate inputs to the dual-stream encoder, which enabled the effective isolation and processing of distinct noise characteristics. The final denoised output was obtained by applying spatiotemporal filtering kernels, as predicted by the network, through per-pixel convolution. The experimental results demonstrate that our method outperformed the existing baseline models across multiple volume-rendering denoising benchmarks in terms of both the visual quality and computational efficiency.

The main contributions of our work are as follows:

- Spatiotemporal VPT Denoising Framework: We propose a flexible framework capable of denoising both single-frame and multi-frame images by effectively combining spatial and temporal information. This approach allows for robust noise reduction across various scenarios.
- Dual-stream Encoder–Decoder Architecture: Our architecture separately processes non-color-related auxiliary features and color-related radiance features. This design leverages low-noise image information more effectively. Additionally, we incorporated gradient loss into the loss function to preserve geometric details and enhance sharpness.
- Multi-head Adaptive Kernel Prediction Module: We introduced a module that predicts adaptive filtering kernels for each pixel. Unlike traditional reprojection algorithms with fixed parameters, our network learns pixel-wise adaptive parameters, enabling better handling of temporal variations and preserving fine details over time.

Our source code is available at https://github.com/Hazelyu27/MC_RDenoiser (accessed on 18 April 2025).

## 2. The Related Work

Various denoising solutions for MC rendering have been proposed and are categorized by processing time into offline, interactive, and real-time denoisers. Real-time denoisers typically process one frame within 30 ms, while offline denoisers exceed 100 ms per frame. Traditional methods often rely on joint bilateral filters or a model regression algorithm, like Spatiotemporal Variance-Guided Filtering (SVGF) [10] and Blockwise Multi-Order Feature Regression [11]. Recently, learning-based approaches have gained prominence. Some methods directly predict pixel values using neural networks [5], while others predict local reconstruction kernels to filter the original image [6]. By leveraging neural networks to estimate the kernel weights, these approaches ensure that predicted colors remain within the convex hull of the input image’s color domain.

### 2.1. Learning-Based Direct Prediction Denoiser

A common approach is to design a network that directly predicts the noise-free radiance of individual pixels. Chaitanya et al. [5] demonstrated this method using multiple frame samples as the input to predict denoised pixel colors. This direct prediction approach is simple to implement and effective at achieving results close to the reference quality, even with low spp images. U-Net-like architectures are often employed for their strong feature extraction and detail preservation capabilities. For instance, Xin et al. [12] proposed a lightweight network with fewer parameters, improving the performance while maintaining fine details. However, U-Net-based outputs can exhibit instability, and random gradients during training may hinder the convergence and optimization [13].

Generative adversarial networks (GANs) are also widely used in image reconstruction due to their ability to capture high-frequency details and generalize effectively. Xu et al. [14] leveraged GANs to learn feature distributions, producing realistic representations of fine details and global illumination. Liu et al. [15] combined adversarial methods with residual structures to extract noise features and spatial dependencies comprehensively. Additionally, ensemble techniques have been explored in MC denoising. Han et al. [16] assessed the importance of auxiliary features and adopted collaborative training strategies, while Xin et al. [12] employed multiple base denoisers with pixel-level ensemble weighting to enhance image fidelity.

### 2.2. Learning-Based Kernel Prediction Denoiser

Bako et al. [6] introduced a kernel prediction network using convolutional neural networks (CNNs) to estimate per-pixel local kernel weights, achieving faster convergence and more stable error gradients. Vogel et al. [17] extended this approach to the temporal domain, delivering high-quality results, even with limited datasets. Recent advancements have combined neural networks with various filters to enhance the effectiveness. For instance, Meng et al. [18] integrated differentiable bilateral grids into their training pipeline, while Isık et al. [13] improved the bilateral filtering by computing range kernels for affinity features to capture high-frequency details. Fan et al. [7] proposed predicting kernel maps instead of per-pixel weights, significantly reducing the network throughput. Additionally, multi-scale structures have been applied to kernel prediction networks. Balint et al. [19] developed a pyramid filter network to effectively reduce low-frequency noise and artifacts, and Chen et al. [20] employed similar cascaded networks. However, for highly noisy data (e.g., 1–2 spp), extracting useful information remains challenging, often resulting in blurred artifacts or blocky predictions.

### 2.3. Denoising for DVR

Iglesias et al. [21] introduced a fitting-based denoising method using weighted recursive least squares to update model parameters. Hofmann et al. [4] employed dual networks to process different features separately, alongside a generative adversarial network to enhance the realism. However, the complexity of this approach hindered the real-time performance. In later work, Hofmann et al. [9] utilized a deep learning denoiser with a U-Net architecture to denoise the rendering results of the medium. While their denoising framework finds utility in sparse volume rendering, scientific visualization demands higher fidelity of details. Bauer et al. [22] proposed FoVolNet, a foveated rendering technique that uses deep neural networks to improve the rendering quality. Taibo et al. [23] developed an immersive 3D medical visualization system, leveraging extended linear regression denoising for real-time cinematic rendering in AR/VR. Xu et al. [8] realized real-time rendering with high quality and temporal stability in VPT-based DVR by developing a decoupling-based denoising technique.

## 3. Methodology

**Figure 1:**
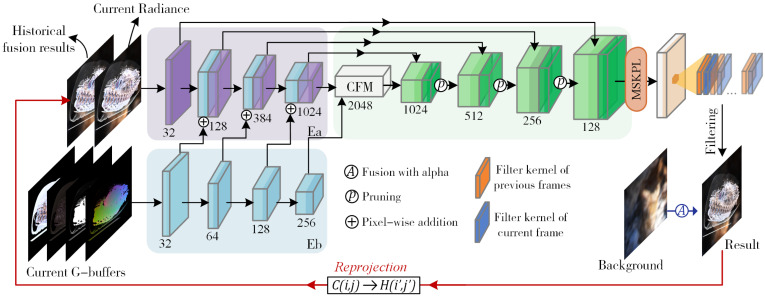
The entire denoising pipeline. Our network includes three stages: (1) The dual-stream encoder separately receives the G-buffers and radiance as inputs and obtains encoded feature maps through the cascade fusion module (CFM). (2) The pixel-wise weight kernel is generated from a multi-head spatiotemporal kernel prediction layer (MSKPL) based on the historical temporal reprojection and current radiance. (3) The decoder receives the fused representation from the CFM, and the decoder module outputs the denoised radiance values. The denoised radiance is then blended with the background to produce the final results.

In this paper, we propose a spatiotemporal kernel prediction network for VPT image sequences to achieve real-time ray-tracing volume-rendering denoising. The overall denoising pipeline is illustrated in Figure 1.

The reprojection algorithm was employed to obtain reprojection information from historical frames. Traditional reprojection techniques utilize the weighted summation of historical and current frame information, where weight calculation is often fixed [24] or based on empirical models [10]. However, due to viewpoint changes, object occlusion relationships, and lighting variations, the reference value of historical frames varies for different pixels within the same scene. To address this, we introduced temporal filtering kernels into the kernel prediction network to adaptively adjust the per-pixel weights during temporal fusion.

Given the distinct value ranges and noise characteristics inherent in radiance features and G-buffer features, we propose a dual-stream convolutional neural network architecture tailored for kernel prediction. In this framework, the reprojected features from historical frames and the radiance features of the current frame serve as inputs to the core encoder, while the G-buffers are fed into the guidance encoder. Unlike traditional unified encoder–decoder structures, our dual-stream encoder exhibits enhanced capability in handling data with heterogeneous noise levels, particularly excelling in deep feature extraction and refinement. Specifically, the G-buffers, which exhibit relatively lower noise levels, and the radiance features, which are inherently noisier, are encoded independently. These intermediate feature maps are subsequently integrated through a cascaded fusion module, effectively decoupling radiance information from non-color related features. The final denoised output is obtained by applying per-pixel filtering using the kernels predicted by the network. To optimize the computational efficiency, we incorporate feature map channel pruning during the training phase. The entire pipeline constitutes an end-to-end trainable architecture, formally represented as a mapping function *g* from *X* to *R*, where *X* = (G-buffers, Radiance). When using neural networks, *N* data pairs $X_{1},X_{2},...,X_{n}$ are input, and the optimal estimated parameters $\hat{\theta }$ are obtained through loss function optimization:

(1) $$\hat{\theta }=min\frac{1}{N}\sum_{i=1}^{N}l\left(X^{i};\theta \right)$$

### 3.1. Dual-Stream Convolutional Encoder–Decoder Network

**Figure 2:**
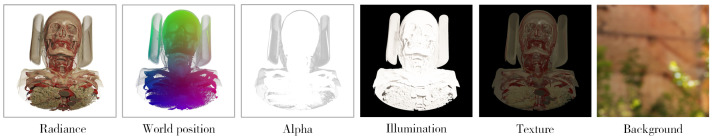
Radiance and G-buffers (world position, alpha, illumination, texture, background) from a single DVR frame.

In the feature map extraction process, a convolutional neural network-based encoder–decoder structure [25] was employed, consisting of two parallel encoders $E_{a}$ and $E_{b}$ and one decoder. Each encoder block comprises two 3 × 3 dilated convolution layers, LeakyRelu layers, and max-pooling layers. There are two primary reasons for separately encoding the input features: First, G-buffers exhibit lower noise levels, facilitating easier extraction of the edge and detail information, while the radiance values obtained from the shading estimation contain higher noise levels [26]. G-buffers are suitable as guidance for denoising radiance features. Second, the aim of our denoiser was to fit the radiance, whereas G-buffer information is color-independent, leading to a reduced network learning efficiency and a color bias in the final output. Therefore, the input section separates the encoding of radiance features from G-buffers, allowing the G-buffer features to be used solely for the connection and guidance between encoders. The G-buffers used in our study contained four channel features: Texture, illumination, alpha, and world position. The radiance and G-buffers are present in Figure 2.

**Figure 3:**
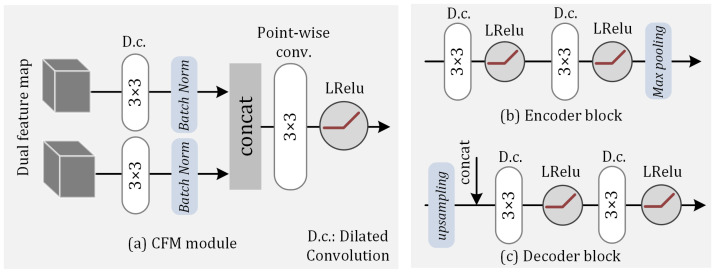
Architectural details of the denoising neural network: (**a**) cascaded fusion module (CFM); (**b**) structure of the encoder block; (**c**) structure of the decoder block.

The outputs of each layer’s radiance encoder block and G-buffer encoder block jointly serve as inputs to the next layer’s radiance encoder block. The low-noise G-buffers can more effectively preserve details during the encoding process and provide guidance for the high-noise radiance encoder. The specific implementation process (Figure 3) is as follows:

1. **Parallel encoding:** Assuming the input feature map has dimensions (W, H), the output of the i-th layer in $E_{a}$ is $x_{i}$, and the output of the i-th layer in $E_{b}$ is $h_{i}$. The computation process is as follows:
  (2) $$h_{i}=LeakyRelu\left(E_{b}\left(h_{i-1}\right)\right)$$
  (3) $$x_{i}=LeakyRelu\left(x_{i-1},maxpool\left(h_{i}\right)\right)$$
2. **Cascade fusion:** The feature maps generated by the dual-stream encoder are fused using our cascade fusion module (CFM). The CFM consists of convolutional layers, batch normalization layers, and pixel-wise convolutional layers to ensure the standardization of the dual-channel feature map distribution and enhance information integration capabilities.
3. **Residual connection and decoding:** Assuming the *i*-th output result of decoder *D* is $d_{i}$, the calculation formula is
  (4) $$d_{i}=D\left(\mathrm{unsample}\left(d_{i-1}\right)⊕x_{L-i}\right)$$
  Here, *L* represents the number of blocks in both the encoder and decoder. In this study, we set L=3.

To enhance the efficiency, each encoder employs two dilated convolutional layers for encoding. The input to each decoder is formed by combining the output from the previous decoder with the corresponding encoder’s output. This design improves the generalization capability of the entire encoder–decoder architecture and reduces the information loss during high-dimensional encoding. Additionally, after connecting each decoder’s output with the encoder, we performed pruning on the convolutional layers to control the number of channels in the output feature maps.

### 3.2. Multi-Head Space-Time Kernel Prediction Module

**Figure 4:**
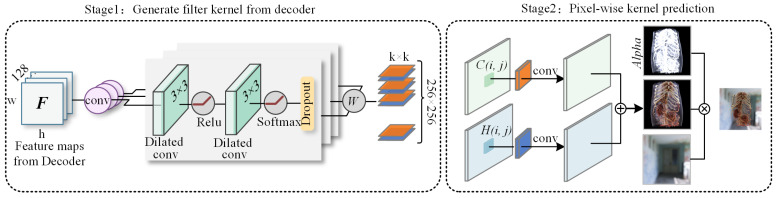
Kernel prediction process for spatiotemporal fusion. Stage 1: The MSKPL module generates two k×k kernels per pixel, representing spatiotemporal neighborhood weight relationships. Stage 2: The predicted kernels perform pixel-wise filtering on both the reprojected historical frame and current frame kernels, followed by alpha blending to produce the final denoised output.

In VPT, due to significant variations in the detail and illumination at different positions in the rendered image, the reference values of historical frames vary across different locations. For instance, in object edge regions, the changes in illumination and shadow between two frames are more pronounced. To address this issue, adaptive temporal fusion can be achieved by learning weight parameters. Similarly, spatial feature fusion can be accomplished through filter kernels in the kernel prediction network. Therefore, this study designed a multi-head spatiotemporal kernel prediction layer (MSKPL), which utilizes a neural network to simultaneously fit two different kernels, $K_{h}$ and $K_{c}$, representing temporal-feature-filtering weights and spatial-feature-filtering weights, respectively. The specific implementation process is shown in Figure 4.

First, the reprojection of the historical frame is computed onto the current frame, resulting in $x_{n}^{p}$, where *n* denotes the *n*-th frame. For the denoised result of the previous frame, it is reprojected onto the current frame using motion vector *v*:

(5) $$x_{n}^{p}\left(i,j\right)=x_{n-1}^{\prime }\left(i+v,j+v,j\right)$$

The reprojection process utilizes the nearest sample point along each viewing ray that satisfies the opacity threshold condition (α>0.8), as determined through ray-marching sampling. In cases where no sample meets this opacity criterion, the algorithm defaults to selecting the sample point exhibiting the maximum opacity value along the corresponding ray [8].

**Figure 5:**
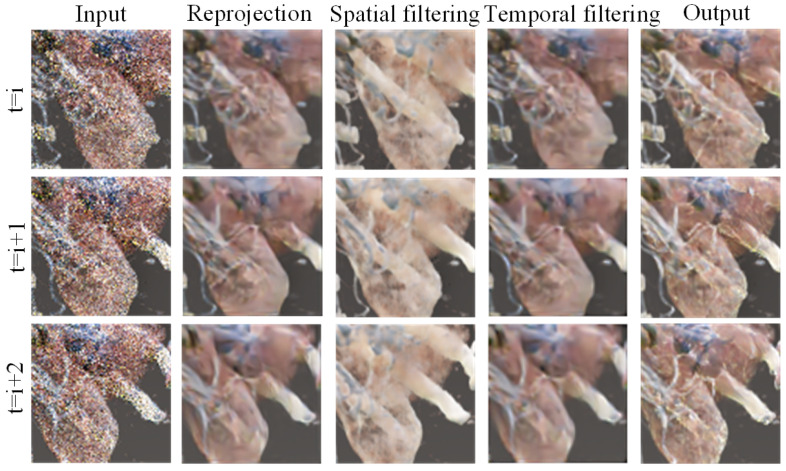
Visualization of intermediate features in temporal denoising process.

Then, our denoiser integrates multiple input sources: The reprojected historical features, current frame radiance values, and corresponding G-buffer attributes. Formulated as a denoising function S, the model generates the denoised output $x_{n}^{\prime }$ for the *n*-th frame through Formula (7):

(6) $$K_{h},K_{c}=S\left(x_{n-1}^{\prime },x_{n},gbuffers\right)$$

(7) $$x_{n}^{\prime }\left(i,j\right)=K_{h}\left(i,j\right)*x_{n}^{p}\left(i,j\right)+K_{c}\left(i,j\right)*x_{n}\left(i,j\right)$$

where $x_{n}$ represents the noisy image, and * denotes the convolution operation. The denoised pixel value at spatial location (i,j) is represented as $x_{n}\left(i,j\right)$, with $K_{h}$ and $K_{c}$ corresponding to the temporal (historical frame) and spatial (current frame) filter kernels, respectively. Due to the use of the softmax activation function in the output layer, some scene images exhibit polarization during training, where the filter kernel values approach 0 or 1 as the number of iterations increases, preventing the learning of effective weights. To address this issue, dropout regularization [27] was added after the softmax layer, effectively avoiding polarization. Based on the generated filter kernels, pixel-wise weighted filtering is performed on the historical frame reprojection result and the current frame result to obtain the denoised volume data. Figure 5 shows the intermediate denoised images obtained at different stages, demonstrating that different detail information is captured during the spatiotemporal denoising process, resulting in a detail-rich denoised result.

Finally, the denoised volume data are weighted and blended with the background according to the alpha map. The alpha map represents the transparency of the volume data at different positions. The fusion formula is as follows:

(8) $$x_{n}^{\prime \prime }=A\left(i,j\right)\times x_{n}^{\prime }\left(i,j\right)+\left(1-A\left(i,j\right)\right)\times B\left(i,j\right)$$

The background fusion is placed as the final step because the volume data contain numerous semi-transparent structures.

Under normal conditions, the spatial and temporal weight kernels gradually converge to appropriate weights through end-to-end training. However, there are two scenarios where historical frames become unusable: First, when the camera undergoes rapid motion, the illumination changes abruptly, or the transfer function varies quickly, the information from historical frames cannot be effectively utilized. Second, in volume rendering with ray tracing, if a ray passes entirely through transparent regions, the historical frame failure rate can be as high as 70%. In this case, since the ray traverses highly transparent areas, the rendered pixel result will also exhibit significant transparency. Visually, the noise level remains relatively imperceptible. Studies have shown that when the temporal correlation between consecutive frames exhibits substantial variation, users’ perception of noise is significantly reduced [28]. Consequently, the current frame’s pixel value is copied to replace the corresponding historical frame value in our method. At this point, the historical frames become effectively non-functional, thereby degenerating the denoising process to rely solely on the spatial denoising of the current frame.

### 3.3. Network Training and Optimization

During the training process, a weighted loss function combining the gradient and Mean Squared Error (MSE) is employed to optimize the model’s parameters. In image denoising tasks, MSE is typically used to measure the pixel-wise difference between the predicted image and the ground truth image, effectively reducing significant noise residuals [3]. When experimenting with denoising datasets, the MSE objective function can efficiently and stably produce smooth images. However, using the MSE alone often results in overly blurred output images, losing some detail and edge information. To address this, the gradient of the image is incorporated into the objective function, allowing the model to focus more on edges and details, effectively preserving sharp features and reducing blurring, as shown in Equation (10):

(9) $$L_{grad}\left(I,R\right)={▽}_{x}I-{▽}_{x}R_{2}^{2}+{▽}_{y}I-{▽}_{y}R_{2}^{2}$$

(10) $$L_{mse}\left(I,R\right)=I-R_{2}^{2}$$

(11) $$L_{total}\left(I,R\right)=w_{grad}\cdot L_{grad}\left(I,R\right)+w_{mse}\cdot L_{mse}\left(I,R\right)$$

In the experiments conducted in this study, since both losses were of the same order of magnitude, a weighted summation approach was adopted to construct the objective function. To ensure that both components contributed equally, the weights were set as $w_{grad}=0.4$ for the gradient loss and $w_{mse}=0.6$ for the MSE loss. The higher $w_{grad}$ improved the visual edge preservation (critical for our multi-scene composite dataset). However, when the $w_{grad}$ was set too high (above 0.5), the image exhibited a slight color distortion for some data.

## 4. Experimentation and Analysis

### 4.1. Dataset

Our denoising dataset was generated using a VPT-based volume renderer [8]. This renderer employed a ray-marching technique to locate the nearest position where the opacity exceeded a specified threshold and recorded the optical properties at that position. The renderer performed sampling and shading calculations on CUDA and utilized 3D-DDA acceleration to achieve an efficient rendering performance.

**Figure 6:**
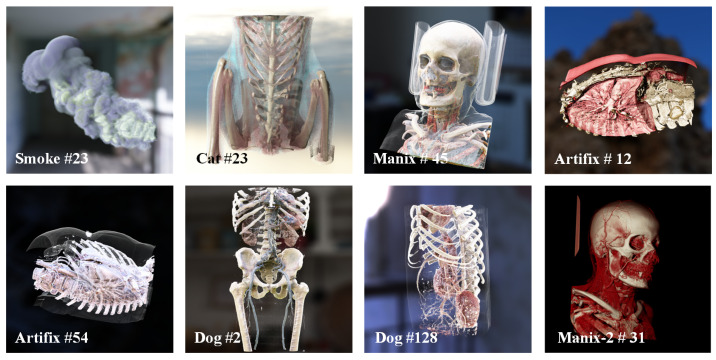
Examples of datasets. The number following # indicates that the image is the k-th frame in the continuous frame image sequence of the dataset.

The dataset comprised eight different volume data, which totaled 65 rendered image sequences. Each sequence contained 320 consecutive frames, with each frame including 1 spp, 2 spp, and 4 spp noisy images; a 4096 spp reference image; and G-buffer features. These image sequences were acquired by recording volumetric data that underwent spiral motion to ensure continuous dynamic changes of the objects from the camera’s perspective. The example data, as shown in Figure 6, covered various spatial resolutions of smoke data, human skull and thoracic CT scans, dog body CT scans, and cat thoracic CT scans. These denoising images showcased a variety of complex translucent structures, such as smoke, blood vessels, and soft tissues, along with their dynamically changing shadows. The rendered images had a resolution of 1024 × 1024 and were stored in 32-bit floating-point format. From the 65 image sequences, 80% were randomly selected for the training set, and the remaining 20% were used for the test set. During the training process, the rendered images were cropped into 256 × 256 image patches, with four patches extracted from each image.

### 4.2. Evaluation Metrics

In our experiment, we employed the Peak Signal-to-Noise Ratio (PSNR) and Structural Similarity Index Measure (SSIM) to estimate the denoising quality of single frames, and used the temporal PSNR (tPSNR) to assess the quality of the multi-frame image sequences.

1. The PSNR is used to quantify the reconstruction quality of images and videos affected by lossy compression. For an input image *I* and a reference image *R*, both with resolution m×n, the PSNR is calculated as follows:
  (12) $$MSE\left(I,R\right)=\frac{1}{mn}\sum_{i=0}^{m-1}\sum_{j=0}^{n-1}{\left[I\left(i,j\right)-R\left(i,j\right)\right]}^{2}$$
  (13) $$PSNR\left(I,R\right)=10\times lg\frac{255}{MSE(I,R)}$$
  where MSE(I,R) represents the Mean Squared Error between the corresponding pixels of the two images. A higher PSNR indicates a better image quality.
2. The SSIM represents the image distortion by detecting changes in the structural information. The SSIM is calculated as follows:
  (14) $$SSIM\left(I,R\right)=\frac{\left(2\mu_{I}\mu_{R}+c_{1}\right)\left(\delta_{IR}+c_{2}\right)}{\left(\mu_{I}^{2}+\mu_{R}^{2}+c_{1}\right)\left(\delta_{I}^{2}+\delta_{R}^{2}+c_{2}\right)}$$
  where $\mu_{I}$ and $\mu_{R}$ represent the mean values of images *I* and *R*, respectively; $\delta_{I}$ and $\delta_{R}$ denote the standard deviations of the two images; and $\delta_{IR}$ represents the covariance between the two images. A higher SSIM indicates a greater similarity between *I* and *R*.
3. For temporal sequences, the tPSNR is used to estimate the stability across consecutive frames [29]. It calculates the difference in the PSNR between the current frame and the previous frame to quantify temporal variations:
  (15) $$tPSNR\left(I,R\right)=PSNR\left(I^{t}-I^{t-1},R^{t}-R^{t-1}\right)$$
  where *t* represents the time index of the current frame.

### 4.3. Model Configuration and Experimental Setup

#### 4.3.1. Model Configuration

The experiments in this study were conducted on a PC workstation using an NVIDIA GeForce RTX 4080 Ti GPU (12 GB VRAM) and an Intel i7-12700KF CPU (32 GB RAM). The denoiser was constructed using the PyTorch (2.0.1) deep learning framework, with acceleration provided by CUDA and cuDNN. The parameter settings were as follows: The Adam optimizer was used with an initial learning rate of 0.0001, and a batch size of 4 was employed to train the network model. Training a complete model took approximately 8 h, and the network parameters converged after around 300 epochs (where the loss decreased to around 0.02).

#### 4.3.2. Experimental Setup

- **Scheme 1—comparative experiments:** Since the proposed method targets real-time denoising tasks for Monte Carlo rendering images, four state-of-the-art methods were selected for the quality and efficiency evaluations: FDAE [4], WRLS [30], SVGF [10], and WSKP [7]. FDAE is a neural network denoising method based on the U-Net architecture; WRLS reduces noise through linear model fitting; SVGF is a spatiotemporal-filtering-based image denoising algorithm that effectively preserves details; and WSKP is a kernel prediction neural network based on shared parameters. For WSKP, the experiment inputs both noisy radiance and auxiliary features (albedo, gradient, depth). In FDAE, in addition to the albedo and gradient, features from the second scattering event are also included as the input. ONND is trained on a large dataset, and its pre-trained model can be directly used for inference.
- **Scheme 2—ablation experiments:** In the ablation experiments, four experiments are designed to verify the impact of different model structures on the denoising results: (1) using the network to directly predict radiance values; (2) using only spatial results with a single encoder–decoder structure; (3) using a parallel dual-encoder–decoder structure; and (4) adding historical frame kernel fusion to the parallel encoder–decoder structure. Additionally, the results for images with different noise levels were evaluated to measure the model’s generalization ability.

### 4.4. Experimental Results and Analysis

#### 4.4.1. Comparative Experiments

**Figure 7:**
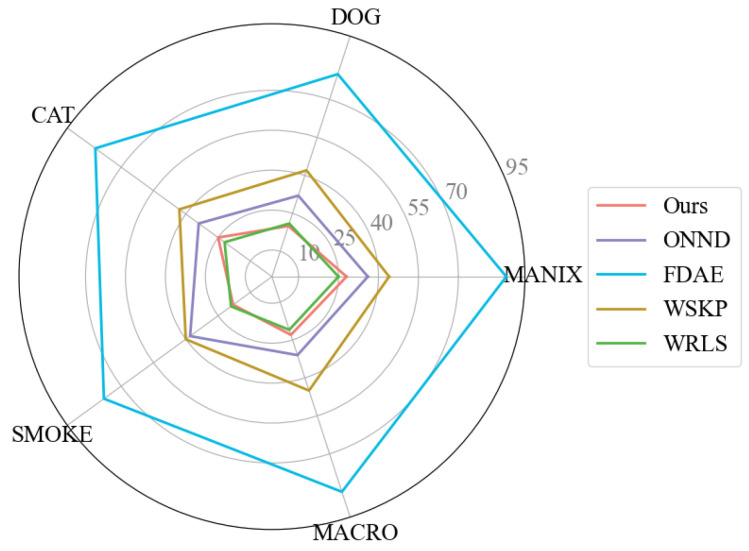
Evaluation of prediction time (ms) from different methods.

The comparative experiments were designed to evaluate the performance of the proposed method against state-of-the-art approaches on three medical CT datasets: A human skull CT, human thoracic CT, and dog thoracic CT. All methods were tested on the same datasets to ensure consistency and fairness. The evaluation metrics were the PSNR, SSIM, and tPSNR, with the results summarized in Table 1. Additionally, the computational efficiency of each method was assessed on five datasets, with the average prediction time after 10 cold-start rounds illustrated in Figure 7.

As shown in Table 1, our proposed method outperformed the other models in both visual quality and evaluation metrics, confirming its feasibility and effectiveness. Specifically, our method achieved an average improvement of 2.3% for the PSNR and 2.16% for the SSIM across the five datasets.

As shown in Figure 7, the prediction time of our method remained below 30 ms per frame across multiple test sets, which was comparable with the WRLS method, and thus, achieved real-time performance. The computational complexity of the denoiser was theoretically determined by the total number of network layers, convolutional kernel size, hidden layer channel dimensions, and downsampling rates, which resulted in an approximately quadratic relationship between the inference time and input resolution. In practical applications, since the resolution of volume data typically does not exceed 512 × 512 × 512, rendering images at 1024 × 1024 resolution is sufficient to reveal the details of the volume data.

**Figure 8:**
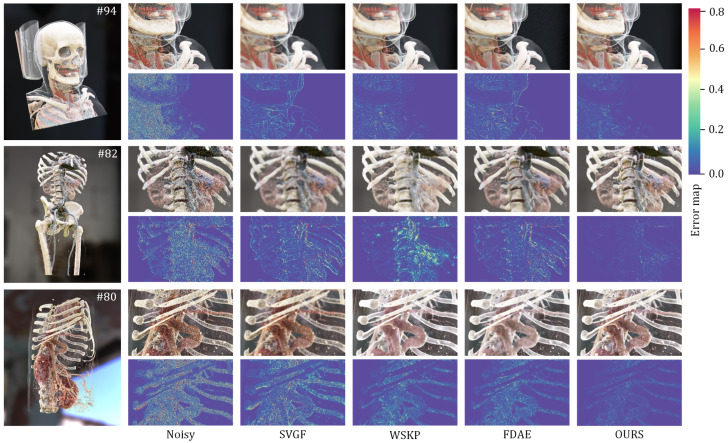
Denoising results and error maps of proposed method and other methods. # indicates frame positions in the sequence.

Figure 8 presents the denoising results and error maps of five different methods. The radiance prediction network (FDAE) demonstrated a detailed texture reconstruction. Notably, FDAE achieved quantitatively superior results on the Manix dataset compared with our proposed approach. The results differ because the approaches are architecturally different. Our denoiser is based on the kernel prediction network, which produces outputs as weighted combinations of neighboring samples. When the shading estimation value contains highlighted outliers (typically related to the scattering distribution function settings), they can significantly impact the denoising results in the MANIX dataset. Since displayed images undergo tone mapping to the [0,1] range, high-noise conditions typically mask such outliers. However, at 800 samples per pixel, these outliers become particularly prominent, with some channel values exceeding 200. The kernel prediction method failed to eliminate these artifacts under such circumstances.

In contrast, FDAE employs a direct prediction network, where outputs rely solely on the network’s direct predictions, enabling effective outlier suppression in such cases. While the direct prediction approach achieves a lower numerical error, it yields perceptually inferior results compared with kernel prediction.

SVGF, on the other hand, tends to introduce artifacts in results, potentially due to the inclusion of non-color attributes in the network input. This suggests that radiance prediction networks struggle to accurately model the nonlinear relationships between multiple features and the output results.

In comparison, WSKP achieved faster denoising times but, as a real-time kernel prediction network with limited receptive fields, it failed to effectively eliminate the high-frequency noise. SVGF adapted spatial filter shapes and sizes by analyzing the image spatial and temporal variance, yet struggled to reconstruct the clear edges in transparent regions of the volumetric data. In contrast, our results more closely resemble the ground truth, showing better handling of the edge information where objects blended with backgrounds while effectively suppressing noise in the tissue structures and bone surfaces within the volumetric data. Our method benefits from a larger receptive field and separately encoded geometric features to prevent color shifts, which enabled superior generalization across multiple scenes. Unlike networks that directly feed all features into a unified encoder, our approach facilitates better recognition of the relationships between different features and the reference image.

#### 4.4.2. Ablation Study

**Figure 9:**
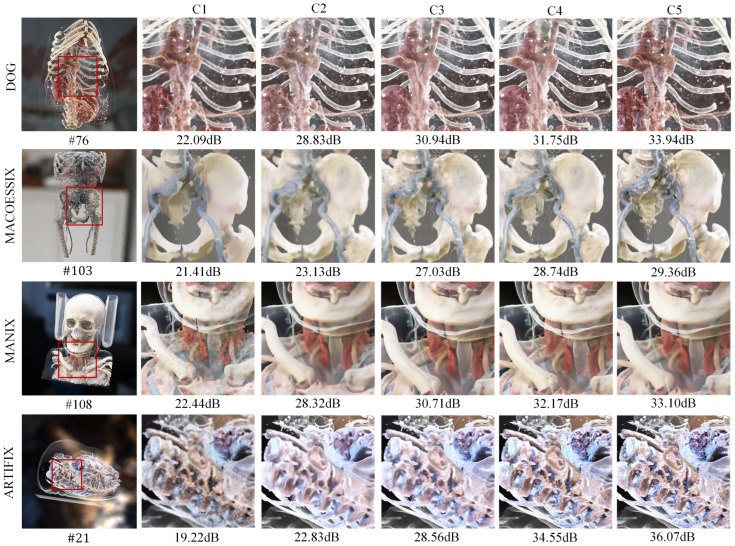
Visualization of ablation experiments.

To verify the effectiveness of the module designs and strategies in the proposed model, this study tested the performance improvements brought by different structures, including five experimental groups: C1, which used an encoder–decoder to directly predict the radiance; C2, which was the spatial denoising result of a single encoder; C3, which was the spatial denoising result of a dual-stream encoder; C4, which was the denoising result without auxiliary features; and C5, which represented the proposed algorithm. The experimental results are shown in Table 2 and Table 3. Figure 9 (C1 to C5) provides a visualization of the different ablation experiment results.

By comparing the results in Figure 9 and Table 2, it can be observed that the dual-encoder architecture significantly enhanced the denoising performance of the model. Compared with the single-encoder approach, the dual-encoder demonstrated superior capabilities in feature separation and deep feature relationship representation, while also exhibiting better performance in modeling radiance features across different frequencies. When employing the dual-encoder in the spatiotemporal domain combined model structure, the PSNR was improved by 4.5 dB and the SSIM value was increased by 0.11 compared with the spatial domain model with a single encoder. The temporal fusion structure effectively enhanced the temporal stability of the network through multi-sample fusion utilizing historical frames. As shown in Table 3, compared with the single-frame sampling method, the fused sampling approach improved the average PSNR by 2.14 dB and the average SSIM by 0.02. These results indicate that the proposed method demonstrated an optimal denoising performance.

#### 4.4.3. Reliability Experiments

**Figure 10:**
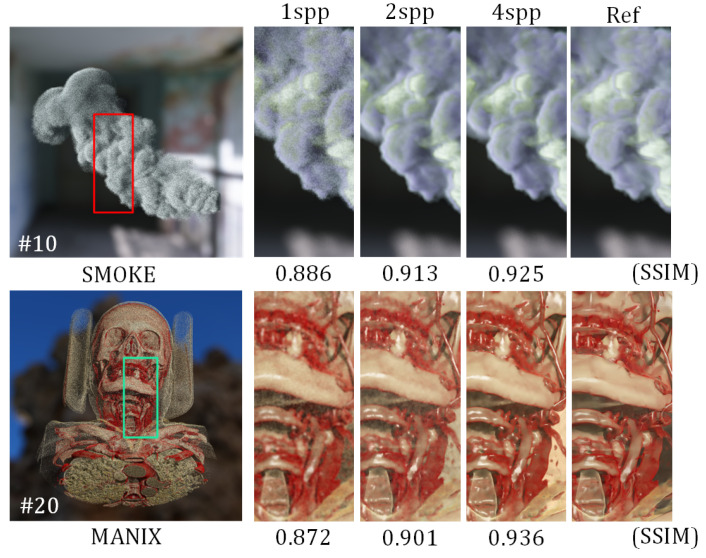
The denoising results using different spp values as the inputs. # indicates frame positions in the sequence.

To evaluate the model’s generalization capability and ensure its adaptability to shadow variations with higher noise levels, we designed experiments to assess the denoising performance under different spp input conditions. The model, trained on 4 spp input images, was tested under 1 spp, 2 spp, 4 spp, and 8 spp conditions. Figure 10 presents the comparative results of our method across various performance metrics under these conditions. Due to the limited effective information contained in 1 spp images, the reconstructed scenes exhibited restricted detail. However, when using rendering images with 2 spp or higher as the input, our method effectively maintained the denoising quality, where it achieved SSIM values that exceeded 0.90 across multiple datasets. Furthermore, the denoising quality showed significant improvement with increasing spp values.

## 5. Conclusions

To address the challenge of real-time denoising for noisy image sequences in volume rendering, we propose a novel temporal-fusion-based neural denoising method for real-time volume rendering. Our approach utilizes a dual-stream encoder to separately encode radiance and geometric features, predicting per-pixel filtering kernels for both historical and current frames to achieve spatiotemporal domain fusion. The network, based on a U-Net framework, employs separate encoding and pruning strategies to achieve a lightweight structure. The denoiser demonstrated strong feature reconstruction capabilities, where it efficiently generated high-quality noise-free image sequences. Comparative and ablation experiments on five test sets show that our method provided superior denoising results compared with existing techniques.

However, our method has some limitations. First, the constraints of the lightweight network structure limited our ability to incorporate complex architectures for capturing dependencies among deep image features, especially for medical imaging requiring the observation of fine lesion areas (e.g., coronary arteries). Therefore, integrating attention mechanisms or similar architectural components could enhance the model’s ability to perceive depth and shadow information. Second, although our algorithm produced visual results that were close to the standard reference on consecutive frames, the lightweight network struggled to fully reconstruct details when processing specific medical structures and materials. Increasing the number of network parameters and incorporating more effective input features could further enhance the final results. Additionally, future work could focus on optimizing the computational efficiency of the algorithm and extending the denoising technology to various real-time applications, such as integrating image segmentation, image enhancement, and view synthesis tasks.

## Figures and Tables

**Table 1:** Comparison of the baseline methods. The denoising quality was measured using the PSNR (dB), SSIM, and tPSNR (dB), with the metrics for each dataset averaged over the test datasets, which consisted of 80 consecutive frames. The best quality score is highlighted in bold.

Methods	Metrics	Manix	Dog	Cat	Artifix	Smoke	Average
	PSNR	37.050	**33.687**	**37.315**	**37.516**	**35.910**	**36.295**
Ours	tPSNR	40.562	**37.824**	**41.331**	**39.003**	**38.100**	**39.364**
	SSIM	0.931	**0.933**	**0.941**	**0.963**	**0.969**	**0.947**
	PSNR	24.212	23.142	25.248	24.902	26.749	24.86
SVGF	tPSNR	27.975	27.724	28.115	27.121	31.543	28.496
	SSIM	0.834	0.861	0.854	0.882	0.861	0.858
	PSNR	**38.521**	31.358	36.098	35.614	35.692	35.456
FDAE	tPSNR	**41.163**	33.534	40.664	35.765	38.122	38.650
	SSIM	**0.937**	0.905	0.910	0.914	**0.969**	0.927
	PSNR	21.741	22.255	23.241	21.962	23.751	22.59
WSKP	tPSNR	25.089	25.741	27.029	25.672	24.010	25.508
	SSIM	0.827	0.793	0.813	0.867	0.873	0.834
	PSNR	28.64	31.081	32.834	30.700	31.842	31.019
WRLS	tPSNR	21.734	34.673	36.339	34.670	34.423	35.175
	SSIM	0.895	0.906	0.913	0.920	0.948	0.916

Bold values indicate the best score in comparison methods.

**Table 2:** Evaluation results of the first two tasks in the ablation study: (1) The impact of the encoder type and scale on the results was assessed using the SSIM, where *K* represents the number of network blocks included in the encoder. (2) The results of using only the current frame as the input were compared with those of temporal denoising utilizing historical frame information.

Datasets	Single Encoder		Dual-Stream Encoder		Denoise for One Frame		Space-Time Fusion	
	**K = 3**	**K = 4**	**K = 3**	**K = 4**	**SSIM**	**PSNR**	**SSIM**	**PSNR**
Artifix	0.791	**0.798**	0.889	**0.916**	0.903	32.704	**0.916**	**34.841**
Dog	0.813	**0.823**	0.872	**0.931**	0.905	32.293	**0.931**	**34.186**
Macro	0.804	**0.852**	0.873	**0.928**	0.896	31.253	**0.928**	**33.341**

Bold values indicate the best score in experiment group.

**Table 3:** The results of the third ablation study task, which measured the difference between using our designed adaptive weight kernel fusion and using a fixed weight kernel for whole-image fusion.

Datasets	Pixel-Wise Weighted		Whole-Image Weighted (w=0.85)		Whole-Image Weighted (w=0.9)	
	**SSIM**	**PSNR**	**SSIM**	**PSNR**	**SSIM**	**PSNR**
Artifix	**0.916**	**34.841**	0.892	30.342	0.887	30.765
Dog	**0.931**	**34.186**	0.923	32.553	0.911	32.439
Macro	**0.928**	**33.341**	0.924	31.300	0.921	31.350

Bold values indicate the best score in experiment group.

## Data Availability

The dataset used during this study is available on the following website: https://www.kaggle.com/datasets/imaginar2t/cbctdata (accessed on 18 April 2025).
